# Factors Influencing Production of Fusaristatin A in *Fusarium graminearum*

**DOI:** 10.3390/metabo5020184

**Published:** 2015-04-01

**Authors:** Anne Hegge, Rikke Lønborg, Ditte Møller Nielsen, Jens Laurids Sørensen

**Affiliations:** Department of Chemistry and Bioscience, Aalborg University Esbjerg, Niels Bohrs Vej 8, 6700 Esbjerg, Denmark; E-Mails: ahegge12@student.aau.dk (A.H.); rlanbo11@student.aau.dk (R.L.); dmni12@student.aau.dk (D.M.N.)

**Keywords:** *Fusarium*, Polyketide synthases, Non-ribosomal peptide synthetases, production, secondary metabolites, mycotoxins, conditions, LC-MS/MS

## Abstract

*Fusarium graminearum* is a ubiquitous plant pathogen, which is able to produce several bioactive secondary metabolites. Recently, the cyclic lipopeptide fusaristatin A was isolated from this species and the biosynthetic gene cluster identified. Fusaristatin A consists of a C_24_ reduced polyketide and the three amino acids dehydroalanine, β-aminoisobutyric acid and glutamine and is biosynthesized by a collaboration of a polyketide synthase and a nonribosomal peptide synthetase. To gain insight into the environmental factors, which controls the production of fusaristatin A, we cultivated *F. graminearum* under various conditions. We developed an LC-MS/MS method to quantify fusaristatin A in *F. graminearum* extracts. The results showed that yeast extract sucrose (YES) medium was the best medium for fusaristatin A production and that the optimal pH was 7.5 and temperature 25–30 °C. Furthermore, production of fusaristatin A was more than four times higher in stationary cultures than in agitated cultures when *F. graminearum* was grown in liquid YES medium. The results also showed that fusaristatin A was only present in the mycelium and not in the liquid, which suggests that fusaristatin A is stored intracellulally and not exported to the extracellular environment.

## 1. Introduction

The *Fusarium* genus comprises several important plant pathogens that are able to produce numerous bioactive secondary metabolites [1]. One of the best studied species is *F. graminearum*, which is a cosmopolitan pathogen of important crops including cereals and maize. The metabolite profile of *F. graminearum* includes the well characterized polyketides aurofusarin, fusarin C and zearalenone as well the recently identified orcinol [2] and fusarielin H [3]. The genome sequence of *F. graminearum* has revealed that it contains 15 polyketide synthases (PKSs) and 19 nonribosomal peptide synthetases (NRPSs) [4,5,6] of which some are conserved throughout the genus while others are specific to *F. graminearum* [7,8].

**Figure 1:**
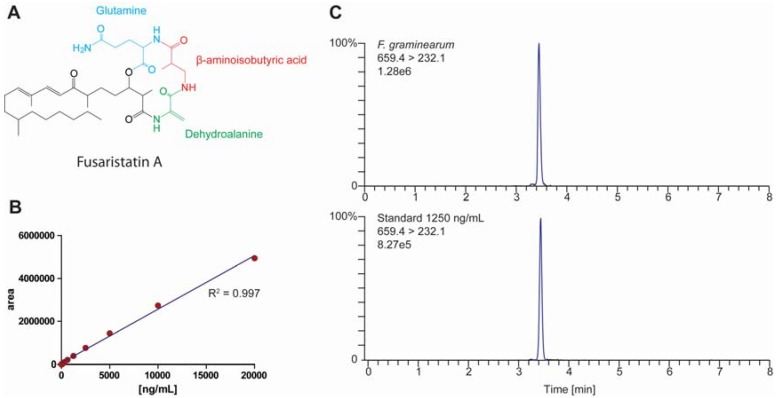
(**a**) Structure of fusaristatin A. (**b**) Standard curve of fusaristatin A in a twofold dilution series (19.5–20,000 ng/mL). (**c**) Chromatograms of fusaristatin A in reference solution (1250 ng/mL) and in an extract from *F. graminearum* grown on yeast extract sucrose (YES) containing 8.8 µg/mL.

Recently the biosynthetic gene cluster for fusaristatin A was identified in *F. graminearum* [9] and *F. avenaceum* [10]. Fusaristatin A is a cyclic lipopeptide consisting of C_24_ reduced polyketide connected to the three amino acids dehydroalanine, β-aminoisobutyric acid and glutamine (Figure 1A). Fusaristatin A was discovered in an unidentified *Fusarium* strain (YG-45) where it had an inhibitory effect against lung cancer cells LU 65 [11]. The compound has later been observed in *F. tricinctum* where it was induced through co-cultivating with *Bacillus subtilis* and *Streptomyces lividans* [12]. This observation is puzzling as fusaristatin A is not active against *B. subtilis* and *S. lividans* [12] or other bacteria including *Staphylococcus aureus*, *S. pneumoniae*, *Escherichia coli, Pseudomonas aeruginosa* and *Enterococcus faecalis* [11,12]. Minor effects against *Xanthomonas oryzae* has however been observed [13].

The biosynthetic gene cluster is predicted to include genes encoding a PKS and an NRPS as well as a cytochrome P450, aminotransferase and a protein with unknown function containing a stress-responsive A/B barrel domain [9]. Biosynthesis of fusaristatin A is proposed to be initiated with production of a reduced polyketide by PKS6. The polyketide is then assimilated by NRPS7 which furthermore incorporates dehydroalanine, β-aminoisobutyric acid and glutamine before fusaristatin A is released by cyclization. The gene cluster is in addition to *F. avenaceum* and *F. graminearum* also present in *F. culmorum* and *F. acuminatum* but not in *F. pseudograminearum* [7], which is a close relative of *F. graminearum*.

Because fusaristatin A was only recently identified in *F. graminearum* nothing is known about the environmental factors which control the production. To identify the effects of some of these factors *F. graminearum* was cultivated under different conditions. The production of fusaristatin A was subsequently examined with a developed LC-MS/MS method.

## 2. Results and Discussion

### 2.1. Quantification of Fusaristatin A

To be able to quantify production of fusaristatin A in *F. graminearum* an LC-MS/MS method was developed, where the optimal parameters were obtained by automatically adjusting the selected reaction monitoring (SRM) settings (Table 1). Fusaristatin A was quantified in positive ionization mode using the protonated [M+H]^+^ ion (m/z: 659.4) as parent ion and three products ions (m/z: 232.1, 303.3 and 359.3) for quantification and verification.

**Table 1:** Parameters for selected reaction monitoring (SRM) transitions for fusaristatin A.

	RT^a^	Precursor ion	Product ions^b^	S-lens	CE^c^
Fusaristatin A	3.45	659.4 [M+H]^+^	232.1/303.3/359.3	121	24/26/22

^a^ Retention time; ^b^ Quantifier/qualifier/qualifier ions; ^c^ Collision energy (V) for product ions

Fusaristatin A ionized very well under the selected conditions, which was reflected in the signal to noise ratios of the three product ions ranging from 13023 to 32153 in the lowest standard concentration (19.5 ng/mL). Fusaristatin A was quantified using the transition 659.4 > 232.1 *m*/*z* in a linear manner (R^2^ = 0.997, Figure 1B) in the standard series (19.5–20,000 ng/mL) as a single peak in the standard series and in the *Fusarium* extract (Figure 1C). This standard curve was used to quantify the fusaristatin A levels in the *F. graminearum* extracts.

### 2.2. Factors Influencing Fusaristatin A Production in Fusarium graminearum

**Figure 2:**
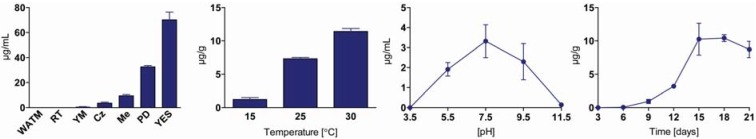
Production of fusaristatin by *F. graminearum* on seven different media, on Cz medium at pH 3.5–11.5, on YES medium after 3–days and on YES medium at 15–30 °C. The experiments were performed with 4–5 replicates and error bars represent standard error of mean (SEM).

To examine whether production of fusaristatin A is influenced by media *F. graminearum* (NRRL 31084) was cultivated in 24 well microtitre plates containing 0.5 mL Wickerhams Antibiotic Test Medium (WATM), Raulin Thom (RT) medium, yeast mold (YM) medium, Czapek dox (Cz) medium, malt extract (ME) medium, potato dextrose (PD) medium and yeast extract sucrose (YES) medium (Figure 2). Fusaristatin A was produced in highest levels on YES, whereas YM, RT and WATM did not support production of fusaristatin A. YES medium is widely used for production of secondary metabolites in fungi [14] and we have previously found this medium superior for production of fusarielins in *Fusarium* [15]. Yeast extracts from different brands can have a huge impact on production of secondary metabolites [16,17] and in the present study we used yeast extract from Scharlau, which we have previously found to be good for production of the polyketides aurofusarin, fusarielin H, fusarin C and zearalenone in *F. graminearum* [16]. The carbon source can also have huge effects on production of secondary metabolites in *Fusarium* [18]. The production of trichotheces has been shown to be induced by sucrose in *F. asiaticum* and *F. graminearum* [19,20]. Furthermore sucrose also supports a higher production of bikaverin in *F. fujikuroi* than glucose [21] and other carbon sources [22].

The effect of pH on fusaristatin A production was examined using microtitre plates with Cz medium adjusted to pH3.5, 5.5, 7.5, 9.5 and 11.5. The highest production of fusaristatin A was observed at pH7.5 (Figure 2), which is closest to the optimal growth conditions for *F. graminearum.* The effect of temperature on fusaristatin A production was measured on YES agar medium at 15 °C, 25 °C, and 30 °C (Figure 2). The highest production of fusaristatin A was observed at 30 °C, which has also been shown as the optimal temperature for production of deoxynivalenol in media with low water activity [23], which is the case for YES medium. The production of fusaristatin A on solid YES medium was examined over time and the first detectable amounts were observed at day 6 (Figure 2). Fusaristatin A continued to accumulate and reached the maximum level (10.4 µg/g medium) after 18 days. The level of fusaristatin A was lower after 21 days, which suggests that the compound was degraded by the *F. graminearum*.

To examine whether the fusaristatin A production is influenced by agitation *F. graminearum* was cultivated in 100 mL liquid YES medium for two weeks at 25 °C. The mycelium was separated from the liquid media to examine whether fusaristatin A is exported to the extracellular environment.

**Figure 3:**
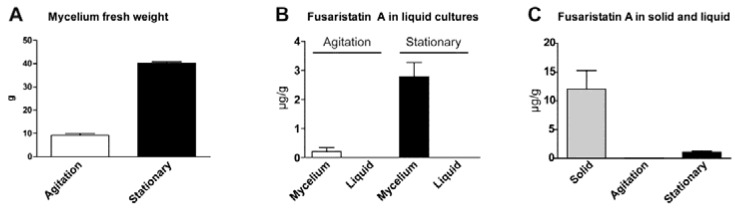
(**A**) Weight of mycelium *F. graminearum* in agitated (90 rpm) and stationary liquid YES medium after incubation for two weeks at 25 °C. (**B**) Production of fusaristatin A in agitated and stationary cultures (µg/g fresh mycelium and lyophilized medium). (**C**) Comparison of fusaristatin A yield in solid and liquid cultures (µg/g cultivation medium). The experiments were performed in triplicate and the error bars represent standard error of the mean (SEM).

The weight of the harvested mycelium was more than four times higher in the stationary cultures (40.2 g), which had grown as a thick surface layer, compared to the agitated cultures (9.1 g), which had it grown as smaller filaments in the agitated medium (Figure 3A). Production of fusaristatin A was 14 times higher in the stationary cultures (2.8 µg/g mycelium) than in the agitated cultures (0.2 µg/g mycelium) (Figure 3B). Although we did not measure recovery rates of fusaristatin A from the solid and liquid, the results clearly show that production of fusaristatin A is highest on solid media. The results showed furthermore that fusaristatin A was only present in the mycelium and not in the medium, suggesting that fusaristatin A is produced and stored intracellularly. This is also supported by analysis of the biosynthetic gene cluster, which is proposed not to contain a transporter [9]. The results also showed that production of fusaristatin A per g YES medium was significantly lower in the liquid cultures (0.02 and 1.11 µg/g medium for agitated and stationary cultures, respectively) than in the solid cultures (12.06 µg/g medium) (Figure 3C). The poor production of secondary metabolites in liquid cultures has previously been noticed in several fungi [24], including *F. graminearum* [25].

## 3. Experimental Section

### 3.1. Chemicals

All chemical solvents were obtained from Sigma-Aldrich (St. Louis, MO, USA). Fusaristatin A was available from a previous study [9] and stored in methanol as a stock solution at −20 °C.

### 3.2. Quantification of Fusaristatin A

A method for quantification of fusaristatin A was developed on a dionex UltiMate 3000 UHPLC system (Idstein, Germany) connected to a Thermo Vantage triple stage quadrupole mass spectrometer (Thermo Fisher Scientific, San José, CA, USA) with a heated electrospray ionization probe. The settings for selected reaction monitoring (SRM) transitions for fusaristatin A were automatically optimized using a 10 µg/mL fusaristatin A solution. The LC was performed with a kinetex phenyl-hexyl column (2.6 μm, 2-mm i.d. × 100-mm, Phenomenex, Torrance, CA, USA) using a constant flow of a 0.4 mL/min and gradient system consisting of A (H_2_O:acetic acid; 99:1) and B (MeCN:H_2_O:acetic acid; 89:10:1), both buffered with 5 mM ammonium acetate. The gradient started at 50% B increasing to 100% over 8 minutes, which was maintained for one minute before reverting to 50% B in one minute and equilibrated for two minutes. The following ion source parameters were used for detection of fusaristatin A: spray voltage (4.5 kV), vaporizer temperature (350 °C), nitrogen sheath gas pressure (18 arbitrary units), nitrogen auxiliary gas pressure (12 arbitrary units), and capillary temperature (270 °C). Argon was used as the collision gas and set to 1.5 mTorr. A two fold dilution series (9.8–20,000 ng/mL) of fusaristatin A was made and used to quantify the samples obtained in the study.

### 3.3. Factors Influencing Fusaristatin A Production in F. graminearum

To examine the production of fusaristatin A in *F. graminearum* (NRRL 31084) on different media we used 24 well microtitre plates containing 0.5 mL of Wickerhams Antibiotic Test Medium (WATM), Raulin Thom (RT) medium, yeast mold (YM) medium, Czapek dox (Cz) medium, malt extract (ME) medium, potato dextrose (PD) medium and yeast extract sucrose (YES) medium [14]. The effect of pH was measured using Cz where pH was adjusted to 3.5, 5.5, 7.5, 9.5 and 11.5 before inoculation. All media contained 0.03% phytagel (Sigma-Aldrich) as previously described [26]. Each well was inoculated with 10.000 spores and incubated for two weeks at 25 °C in the dark. The experiments were performed with four replicates. The content of each well was then transferred to a 10 mL glass tube and extracted with 1 mL extraction solution (79% acetonitrile, 20% H_2_O, 1% acetic acid) in an ultra-sonic bath for 45 minutes. The extracts were centrifuged for 1.5 minutes at 12,000 rpm in a microcentrifuge tube and then transferred to 2 mL HPLC vials.

Accumulation of fusaristatin A over time was performed using YES agar medium in 90 mm Petri dishes with three replicates for each time point (3, 6, 9, 12, 15, 18, 21 days). The plates were sliced in approximately 5 × 5 mm squares and extracted with 50 mL extraction solution in a 100 mL glass bottle as described above.

To compare whether production of fusaristatin A is different on liquid and solid media, *F. graminearum* was cultivated in triplicate for two weeks at 25 °C on 90 mm solid YES agar plates and in 100 mL liquid YES medium in 250 mL Erlenmeyer flasks with (90 rpm) and without agitation. The mycelia from the liquid cultures were separated from the media by filtering through Miracloth and the liquid media was subsequently lyophilized. The different samples were weighed and extracted with 50 mL extraction solvent.

All the samples obtained in the study were analyzed with the developed LC-MS/MS method where 3 µL where injected and fusaristatin A quantified using the transition 659.4 > 232.1 *m*/*z*.

## 4. Conclusions

In the present study, an LC-MS/MS method for quantification of fusaristatin A was developed and used to examine the production in *F. graminearum* under different growth conditions. The optimal medium for production of fusaristatin A was YES medium and the optimal pH was 7.5 and temperature 25–30 °C. Furthermore we found that fusaristatin A was present in mycelium and not in the medium, which suggests that it is stored intracellularly and not exported to the extracellular environment.
